# Optimized Spiral Metal-Gallium-Nitride Nanowire Cavity for Ultra-High Circular Dichroism Ultraviolet Lasing at Room Temperature

**DOI:** 10.1038/srep26578

**Published:** 2016-05-25

**Authors:** Wei-Chun Liao, Shu-Wei Liao, Kuo-Ju Chen, Yu-Hao Hsiao, Shu-Wei Chang, Hao-Chung Kuo, Min-Hsiung Shih

**Affiliations:** 1 Department of Photonics and Institute of Electro-Optical Engineering, National Chiao Tung University (NCTU), Hsinchu 30010, Taiwan; 2 Research Center for Applied Sciences (RCAS), Academia Sinica, Taipei 11529, Taiwan; 3 Department of Photonics, National Sun Yat-sen University (NSYSU), Kaohsiung 80424, Taiwan

## Abstract

Circularly polarized laser sources with small footprints and high efficiencies can possess advanced functionalities in optical communication and biophotonic integrated systems. However, the conventional lasers with additional circular-polarization converters are bulky and hardly compatible with nanophotonic circuits, and most active chiral plasmonic nanostructures nowadays exhibit broadband emission and low circular dichroism. In this work, with spirals of gallium nitride (GaN) nanowires (NWRs) covered by a metal layer, we demonstrated an ultrasmall semiconductor laser capable of emitting circularly-polarized photons. The left- and right-hand spiral metal nanowire cavities with varied periods were designed at ultraviolet wavelengths to achieve the high quality factor circular dichroism metastructures. The dissymmetry factors characterizing the degrees of circular polarizations of the left- and right-hand chiral lasers were 1.4 and −1.6 (±2 if perfectly circular polarized), respectively. The results show that the chiral cavities with only 5 spiral periods can achieve lasing signals with the high degrees of circular polarizations.

Circularly polarized light and chiroptical effect^1^ have received considerable attention in advanced photonic and electronic technologies including optical spintronics^2^, quantum-based optical information processing and communication^3^,^4^, and high-efficiency liquid crystal display backlights^5^. Moreover, the development of circularly polarized photon sources has played a major role in circular dichroism (CD) spectroscopy, which is important for analyses of optically active molecules^6^,^7^, chiral synthesis^8^,^9^ in biology and chemistry, and ultrafast magnetization control^10^,^11^. A reliable circularly polarized light-emitting device is therefore of great interest in many fields. However, the conventional collocation of light-emitting devices and additional circular-polarization converters that produce circularly polarized beams makes the setup bulky and hardly compatible with nanophotonic devices in ultrasmall scales. In fact, the direct generation of circularly polarized photons may simplify the system integration, compact the setup, lower the cost of external components, and perhaps enhance the power efficiency.

Various effects of plasmonic metamaterials and related usages^12^ such as negative refraction^13^, extraordinary transmission^14^, cloaking^15^, and quarter-wave plates^16^ have been investigated. One of these remarkable phenomena is the plasmonic chirality, which can control the right- (R-) and left- (L-) hand photon emissions for various applications including circular filters, reflectors, lasers, and biosensors^17^,^18^,^19^,^20^. Most plasmonic chiral metamaterials exhibit considerable energy loss due to the significant absorption of metals. This, on the other hand, leads to the larger dissymmetry factor *g*_e_ of these artificial materials than those of the natural ones (*g*_e_ = ±2 if completely circularly polarized), featuring the higher degree of circular polarizations. In fact, enhancing the dissymmetry factor beyond those of natural molecules by 2 to 3 orders of magnitude through designs of chiral metamaterials is the main objective of the area. For future applications in integrated biophotonic circuits, highly circularly polarized photon sources with a significant power density and small footprint are useful. Although a precursor of such devices could be implemented with conventional semiconductor lasers in conjunction with a linear polarizer and quarter-wave plate, analogous constructions become challenging and impractical as the active device is shrunk to the nanoscale. By directly incorporating the chiral characteristics into active nanophotonic devices, we may control the handedness of output photons in the more straightforward manner.

In this work, with fabricated spirals of gallium nitride (GaN) nanowires (NWRs) covered by thin layers of insulators and metal as active cavities, we demonstrated a semiconductor laser capable of emitting circularly-polarized photons. The chirality was built into these photonic devices through the spirals, and depending on their orientations, the cavities were classified as left- and right-hand chiral (L- and R-chiral) structures. These lasers had small cavity volumes and directly output circularly polarized waves. High dissymmetry factors of about 1.4 and −1.6 were observed in single L- and R-chiral devices with a diameter of 10 μm, respectively. Due to its high degree of circular polarizations and compactness, the chiral light-emitting device may be promising for applications in biotechnology on chips.

## Mode Patterns in Metal-GaN Nanowire Cavities

Many artificial chiral structures such as the gammadion, twisted arc, and spiral have been fabricated as polarization rotators, circular polarization filters, and analyzers^17^,^18^,^19^,^20^,^21^,^22^,^23^,^24^,^25^,^26^,^27^. However, most of these nanostructures are broadband during the operation and do not support high CD due to the broad band operation. In this study, we folded metal-GaN NWRs into spiral laser cavities and their mirror images to achieve compact, high efficiency, single-wavelength light sources with high CD in the ultraviolet (UV) range. Metal nanostructures and nanocavities have been demonstrated for ultracompact lasers^28^,^29^,^30^,^31^,^32^,^33^,^34^,^35^,^36^,^37^. The field confinement due to surface plasmon effect, which differs from and is much stronger than the counterpart resulted from the index contrast in dielectric cavities, can be utilized in metal nanocavities to reduce modal volumes and realize subwavelength-scale lasers. Due to the low absorption of aluminum (Al) in the UV range, it was chosen as the metal layer that supports surface plasmon waves around the metal-GaN interface.

**Figure 1:**
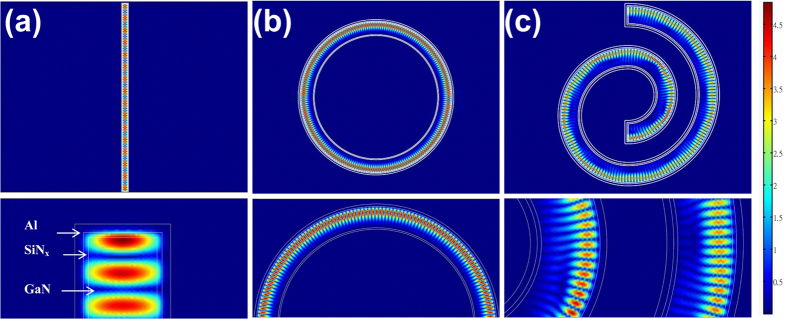
Intensity profiles of metal-GaN NWRs in the forms of (**a**) a straight cavity, (**b**) nanoring, and (**c**) L-chiral structure at a wavelength of 364 nm.

To grasp the field patterns in NWR cavities, we used the finite-element method to simulate them. The field intensity profiles in various types of Al-GaN NWRs are shown in Fig. 1(a–c). Hybrid surface plasmon polariton (SPP) waves are present in these NWR structures. The optical intensity is confined within the GaN core, and the counter-propagating waves along the waveguide form the standing-wave pattern inside these cavities. Several losses may come along with these field patterns. For our chiral NWR cavities, the fractional loss *A* per optical cycle, which is the inverse of total quality (*Q*) factor *Q*_Total_, can be approximated as


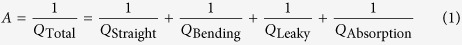


where *Q*_Straight_, *Q*_Bending_, *Q*_Leaky_ and *Q*_Absorption_ are *Q* factors due to the losses from the propagation along the NWR, waveguide bending, leakage towards the GaN substrate (also a minor portion towards the free space of air), and the absorption in the metal and the semiconductor, respectively. The inverses of these *Q* factors characterize the corresponding contributions to the loss *A*.

Figure 1(a) illustrates the top view of a well-confined mode profile in a straight Al-GaN NWR cavity. In the absence of bending, the field profile is undistorted, and the mode experiences no loss associated with it. The bending loss is a major concern whenever the device is twisted. In a curved dielectric NWR at optical frequencies, it could be large if the curvature radius is in the micron range. This issue, however, is less serious in metal NWRs due to the more robust confinement of surface plasmons^38^,^39^,^40^,^41^. The effective refractive index difference between the top GaN nanowire and the bottom GaN substrate is also relative small, which leads to weaker vertical confinement and the vertical leakage in the GaN nanowire cavity. Similar to the bending loss, the power leakage is less stringent in metal structures since photons are not rapidly released from SPP waves into open spaces. In fact, later estimations based on the sizes of realistic devices in the experiment indicate that the joint *Q* factor 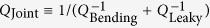 associated with the bending and leakage losses could be high. Thus, ultrasmall nanowire laser cavities have been achieved without much concern on the bending and leakage losses^42^,^43^.

Figure 1(b) shows the field distribution of a well-confined, high-*Q* resonant mode in an Al-GaN NWR cavity whose length is the same as that of the straight one in Fig. 1(a). For circularly symmetric structures, photon emissions with high CD are not easily observed due to the degeneracy in clockwise and counterclockwise rotating waves. The chirality breaks this degeneracy, and therefore the chiral NWR structure is the more favorable scheme in view of a high *Q* and high CD laser cavity with compact geometry. In Fig. 1(c), we also show the field pattern of one resonant mode in the R-hand chiral Al-GaN NWR cavity whose length is the same as those in Fig. 1(a,b). The mode is composed of hybrid SPP waves and can be a promising candidate that provides the single-wavelength lasing output with high CD.

## Fabrication and Experiment

**Figure 2:**
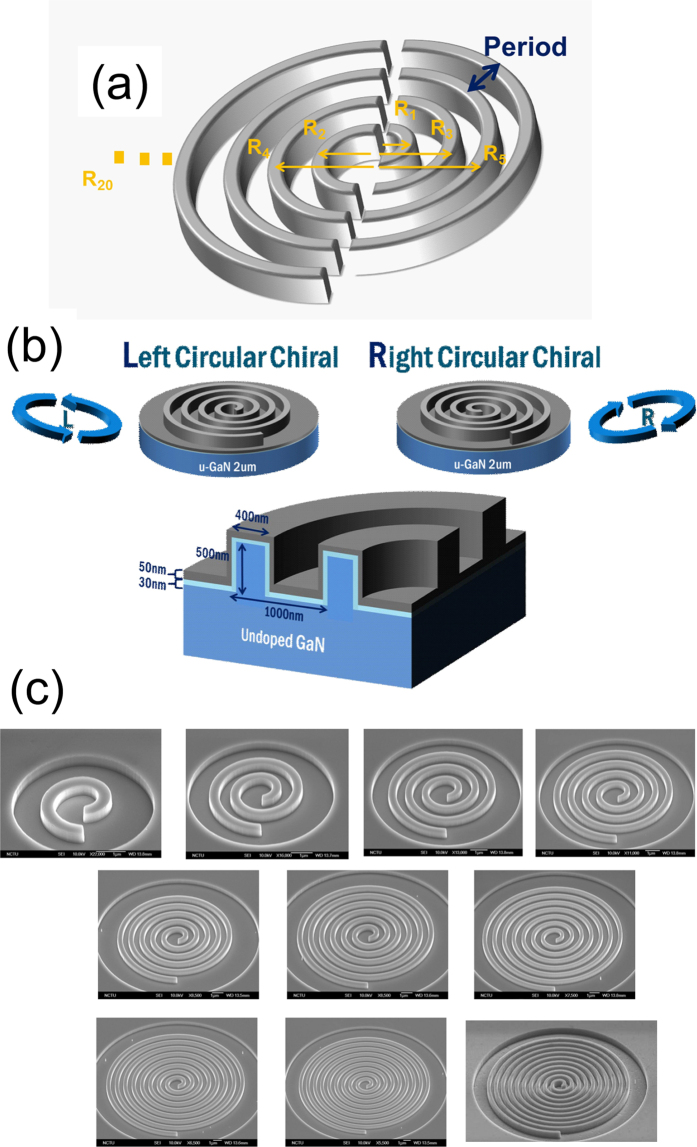
(**a**) Formation of a metal-GaN NWR spiral cavities based on half circular rings with increasing radii. (**b**) Schema of the L- and R-spiral cavities of metal-GaN NWR and their cross section. (**c**) SEM images of the chiral NWR cavities with 1 to 10 periods.

In this study, to obtain optical modes with higher CD, we constructed spiral metal NWR cavities by connecting numerous half nanorings with increasing radii, as shown in Fig. 2(a). The lasing wavelength of the optical modes in the L- and R-hand chiral cavities would be set to match the gain spectrum of the GaN epitaxial layer.

The chiral structures were fabricated on a 2 μm thick undoped GaN layer which was grown on a c-plane (0001) sapphire substrate through metal-organic chemical vapor deposition and played the role of gain medium. Figure 2(b) shows the schemes of L- and R-hand chiral NWR structures fabricated as follows^1^. A 300-nm thick silicon-nitride (Si_3_N_4_) layer that acted as an etching mask was deposited on GaN through plasma-enhanced chemical vapor deposition^2^. Polymethylmethacrylate (PMMA, 250-nm thick) was spin coated on the Si_3_N_4_ layer^3^. The chiral patterns were defined on the PMMA layer through e-beam lithography followed by reactive ion etching using a CHF_3_-O_2_ mixture to etch down to the Si_3_N_4_ layer^4^. The chiral pattern on the Si_3_N_4_ layer was transferred to the undoped GaN layer (around 500 nm in depth) through inductively coupled plasma reactive ion etching using a Cl_2_-Ar mixture. The Si_3_N_4_ mask layers were removed through wet etching^5^. A 30-nm Si_3_N_4_ layer was deposited on the patterned GaN layer^6^. Finally, a 50-nm Al layer was coated on the device through e-gun evaporation to form the chiral structures of metal-coated GaN lasers. We set the radii of half rings as an arithmetic progression of up to 20 terms, with a leading term of 1 μm and a common difference of 500 nm. The width and height of the structure were 400 and 500 nm, respectively. To differentiate the two chiral structures, we term the spiral that rotated clockwise from the inner to the outer as the R-hand chiral structure. The corresponding image across a vertical mirror plane was termed as the L-hand chiral structure. Figure 2(c) shows the scanning electron microscopy (SEM) images of the Al-GaN L-hand chiral structures with numbers of spiral periods up to ten.

## Results and Discussions

**Figure 3:**
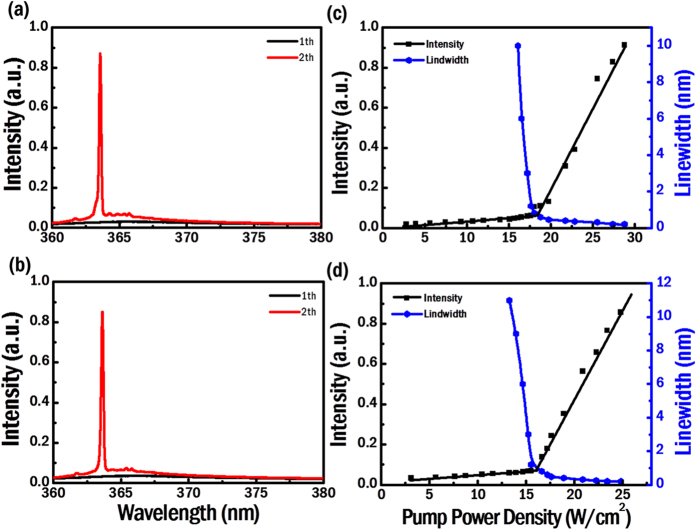
Emission spectra of the (**a**) L- and (**b**) R-hand chiral structures below (black) and above (red) the threshold. The L–L curves (black) and the linewidths (blue) versus the pump power density for the (**c**) L- and (**d**) R-hand chiral lasers.

To characterize the Al-GaN NWR chiral structures with different numbers of periods, we optically pumped the devices using a frequency-tripled Nd:YVO_4_ 355-nm pulsed laser at room temperature. The L- and R-hand chiral cavities with different periods were investigated under the same pumping condition. Figure 3(a,b) shows emission spectra of the L- and R-hand chiral cavities with 10 spiral periods above (red) and below (black) the threshold at room temperature. A lasing peak at a wavelength of approximately 363.5 nm was observed for both cavities. Figure 3(c,d) shows the light-in-versus-light-out (L-L) curves of the lasing modes in the two NWR cavities and their linewidths under different pump power densities. The threshold power densities of L- and R-hand chiral cavities were approximately 17 and 16 W/cm^2^, respectively. The linearity of L-L curves above the threshold indicated the lasing behavior of the two chiral cavities. Moreover, the significant linewidth narrowing around the threshold confirmed that the spontaneous emission of photons in Al-GaN cavities turned into stimulated emission. The experimental quality factor (*Q*) of the spiral metallic nanowire cavity is approximately 450 by estimating the ratio of wavelength to linewidth at transparence. These phenomena showed that the lasing action of chiral Al-GaN NWR cavities could take place at room temperature. The similar lasing characteristics of the L- and R-hand structures indicated that they were *partners* in terms of mirror images.

**Figure 4:**
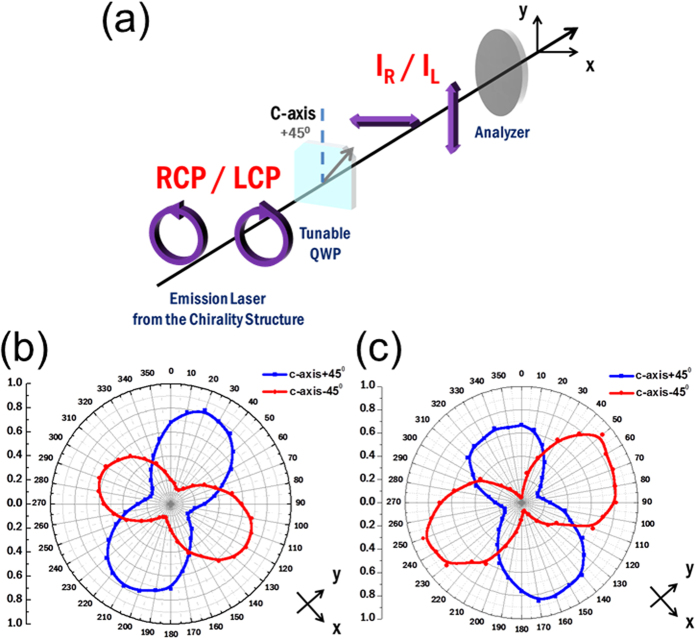
(**a**) The setup of the circular-polarization analyzer that can separate components of the LCP and RCP. The degree of circular polarization could be assessed through the horizontally- and vertically-polarized intensities after the input beam was converted from the quarter-wave plate. Polar plots of the intensities filtered by the linear polarizer versus the polarization angle for the (**b**) L- and (**c**) R-hand chiral lasers.

To investigate the polarizations of the chiral Al-GaN NWR lasers, we inserted a two-stage circular-polarization analyzer in our setup of micro photoluminescence to characterize polarization states of the lasing signals from the L- and R-hand chiral cavities. The circular-polarization analyzer was made up of a tunable quarter-wave plate, in which a *π*/2 phase retardation can be tuned in the wavelength range from 150 nm to 6 μm, and a linear polarizer working in the UV regime. Figure 4(a) shows the setting of circular-polarization analyzer in which the quarter-wave plate was tuned to 364 nm. The axis of the extraordinary refractive index (*c* axis) of the quarter-wave plate was laid at an angle of +45° with respect to the vertical direction (*y* axis). This setup would convert two circular polarizations into two orthogonal linear polarizations. After a beam passed through the quarter-wave plate, the resulted square magnitude of the horizontal component (*x* axis) represented the intensity *I*_R_ of the right-hand circular polarization (RCP) in the input beam. Similarly, the counterpart of the vertical component (*y* axis) indicated the intensity *I*_L_ of the left-hand circular polarization (LCP) in the same beam. If the angle between the *c* and *y* axes was set to −45°, the intensities represented by the horizontally- and vertically-polarized components would be switched to *I*_L_ and *I*_R_ of the incident beam, respectively. The polarization direction of the linear polarizer could be rotated arbitrarily in the *x*-*y* plane and was utilized to filter out the horizontally- and vertically-polarized components of the converted beam, from which the two intensities *I*_R_ and *I*_L_ were deduced. By comparing *I*_R_ and *I*_L_, we can assess whether the polarization state of the laser emission is RCP-like or LCP-like. The dissymmetry factor *g*_e_ which characterizes the degree of circular polarizations can then be evaluated from *I*_R_ and *I*_L_ as follows^40^:


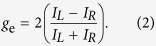


The input beam would be perfectly circularly-polarized (either *I*_R_ = 0 or *I*_L_ = 0) if the magnitude |*g*_*e*_| is 2, which is also the maximum of this quantity. If the magnitude |*g*_*e*_| is closer to 2, the lasing signal would more closely resemble a purely circularly-polarized beam more.

Figure 4(b,c) shows polar plots of the intensities filtered by the linear polarizer versus its polarization angle (referenced to an axis tilted at −45° from the *y* axis) for lasing signals from the L- and R-hand chiral cavities, respectively. The blue and red lines corresponded to measurements as the *c* axis was tilted at +45° and −45° from the *y* axis, respectively. From the blue line in Fig. 4(b), the more dominant vertically-polarized (*y*-polarized) intensity indicated that the emission from the L-chiral cavity was LCP-like. Similarly, from Fig. 4(c), the more significant horizontally-polarized (*x*-polarized) intensity indicated by the blue line revealed that the emission from the R-chiral cavity was RCP-like. By measuring vertically- and horizontally-polarized intensities of the converted beam, we obtained the intensities *I*_L_ and *I*_R_ of the circularly-polarized components in the lasing signals. Subsequently, the dissymmetry factors *g*_e_ of the L- and R-chiral lasers with 10 spiral periods were estimated to be approximately 1.4 and −1.6, respectively. The signs and magnitudes of *g*_e_ confirm that the L- and R-hand chiral cavities produced high fractions of LCP and RCP components in lasing signals, respectively. These high dissymmetry factors are close to theoretical limits corresponding to purely circularly polarized waves (±2) and indicate that the ultracompact, low-threshold laser devices can serve as single-wavelength photon sources with high CD in many applications.

**Figure 5:**
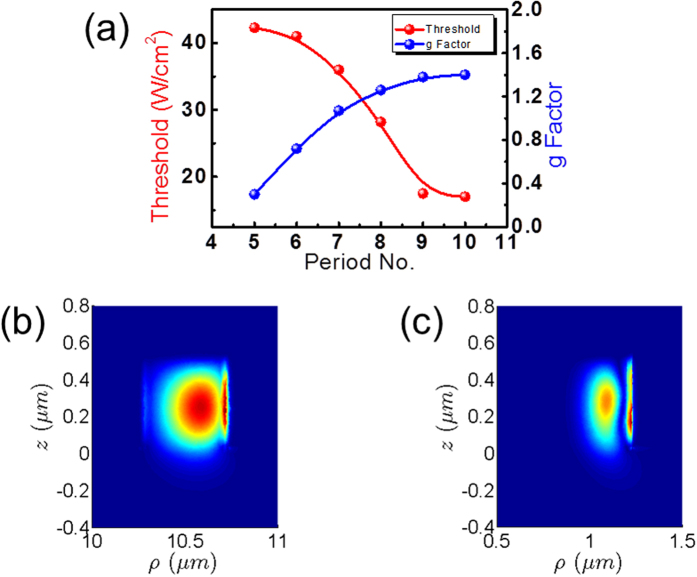
(**a**) Threshold power density (red) and dissymmetry factor *g*_e_ (blue) of the spiral Al-GaN NWR cavities versus the number of periods. The modal profiles of the main filed component |*E*_*ρ*_(*ρ*)| on the cross sections at the (**b**) outer (radius of 10.5 μm) and (**c**) inner (radius of 1 μm) spirals of the 10-period NWR cavity, respectively.

To further understand the size dependence of spiral cavities, the lasing properties of cavities with different numbers of periods were characterized. Spiral cavities which had the same NWR cross section but 1 to 10 periods of half rings with increasing radii were fabricated. The lasing action could be achieved if the number of periods was larger than 5. The lasing thresholds of spiral cavities with 5 to 10 periods are represented by the red curve in Fig. 5(a). The threshold decreased as the number of periods increased. This behavior was attributed to the lower optical loss in the larger spiral cavities because the average bending and leakage losses in the larger cavity were smaller, and the mirror loss at two ends of the chiral cavity became less critical. We have utilized a formulation based on the concept of gain and sources^44^,^45^ to estimate the joint *Q* factor *Q*_Joint_ due to the bending and leakage losses of the chiral cavities. This approach had been applied to realistic active photonic structures with satisfactory matches^43^,^46^. More details about the approach are presented in Method (see Modeling). Using this formulation, we obtained numerical modal profiles of the dominant field component |*E*_*ρ*_(*ρ*, *z*)| (polarized component parallel to the GaN substrate) on the cross sections corresponding to the outer (radius of 10.5 μm) and inner (radius of 1 μm) spirals of the 10-period NWR cavity shown in Fig. 5(b,c), respectively. The wavelength was set to 364 nm. Both modal profiles show slightly leaky fields toward the GaN substrate, suggesting that the leakage is not a serious issue on lasing. On the other hand, the modal profile in the inner spiral is squeezed more closely to the sidewall at the large-radius side due to the smaller curvature radius of 1 μm. Therefore, it is expected that the bending loss would be more significant in the inner spiral, and hence the corresponding *Q*_joint_ would be lower. Indeed, we obtained numerical joint *Q* factors *Q*_joint_ of about 1023 and 408 for the outer and inner half rings, respectively. Still, these joint *Q* factors are high, indicating that the threshold of these chiral lasers could be reached, which is consistent with the lasing phenomena observed in the experiment.

We also characterized the dissymmetry factors *g*_e_ of these spiral cavities and showed them as the blue curve in Fig. 5(a). This factor increased from 0.25 to 1.6 as the number of periods increased from 5 to 10, indicating that the stronger CD was induced from resonant modes in the spiral cavity with the longer cavity length. The degrees of circular polarizations, on the other hand, were not directly related to the rapidly oscillatory standing wave patterns along the NWR cavity shown, for example, in Fig. 1(c). These spatially fast-varying patterns were featured by an effective *in-plane* propagation constant much *greater* than that of the free space (air), and hence the associated fields could not easily propagate normally from the GaN surface towards the free space. In fact, our measurement system detected the highly directive surface emissions normal to the GaN surface (within 5° around the surface normal) from the far-field characterizations of these chiral lasers. This fact also supports that the degrees of circular polarizations and dissymmetry factors *g*_e_ were not closely connected to how the oscillatory profile (near field) was distributed as the number of periods increased.

**Figure 6:**
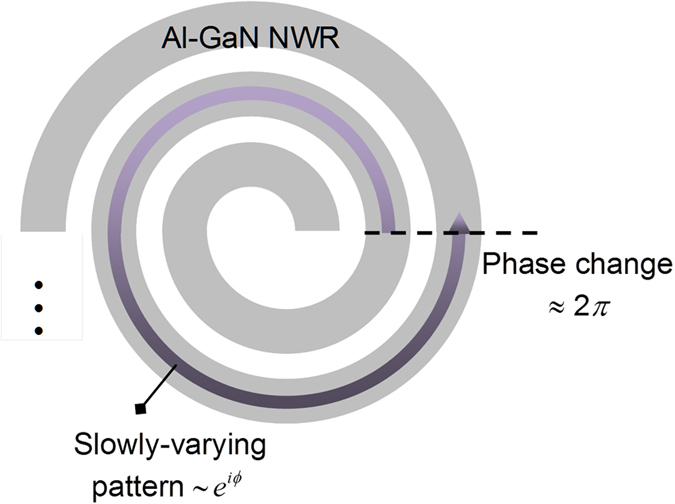
The induced slowly-varying pattern which gains a phase change of about 2*π* for *E*_*z*_(r) and *H*_*z*_(r) after turning 360° in an L-hand chiral NWR. It would have an azimuthal dependency similar to exp(*iϕ*).

We speculate that the circularly-polarized emissions normal to the GaN surface were generated by various minor patterns which were induced by the changes of curvature radius and exhibited spatially slow in-plane variations along the NWR. These minor patterns mainly oscillated within the cross sections of Al-GaN NWRs and hence propagated little along the NWRs. Let us consider an L-hand chiral NWR shown in Fig. 6. If some of the field patterns happened to exhibit a phase change of about 2*π* for *z*-polarized components of the electric field *E*_*z*_(**r**) and magnetic field *H*_*z*_(**r**) after turning 360° along neighboring half rings, they would carry an azimuthal dependency similar to exp(*iϕ*) [exp(−*iϕ*) for R-hand chiral structures]. Only the near fields with azimuthal dependencies exp(±*iϕ*) could be radiated normally from the GaN surface to the far field zone, and their far field behaved just as circularly-polarized waves. Other azimuthal components were either barely radiated to the free space or would propagate to the far-field zone obliquely from the surface. In fact, this criterion is also the condition that determines the minimal cutoff frequency of the spiral antennas which radiate circularly polarized waves at radio frequencies.

These specific slowly-varying patterns were more likely to be induced if the fractional changes of curvature radius between neighboring half rings were significant. Therefore, they tended to appear around the first few inner spirals, got radiated coherently toward the far zone, and formed the highly directive surface emission due to constructive interference analogous to the effect of antenna arrays. As the number of periods first increased, more slowly-varying patterns of this type were induced, and that explains the initial enhancement of the dissymmetry factor *g*_e_. However, the fractional changes between the half rings which were added latter gradually decreased and could no longer induce the required patterns efficiently. This incapability would mark the saturation of *g*_e_ as the number of periods increased, which, according to the experimental data shown in Fig. 5(a), took place around period 8 or 9.

Several groups have studied circularly polarized light-emitting devices with the chirality. Konishi *et al.*^21^ showed that semiconductor planar chiral nanostructures had a *g*_e_ of about −0.528^21^. Maksimov *et al.*^19^ demonstrated semiconductor planar chiral-structured microcavities with *g*_e_ around +1.04 and −0.94^19^. Compared with the CD of previously reported structures, our Al-GaN NWR chiral cavities exhibited the even higher CD with a single-wavelength output and small footprint due to the robust optical confinement and low bending and leakage losses, which are attributed to the effect of surface plasmons in our structures. In this way, our device could directly produce photons with a high degree of circular polarizations from one single compact device. It is worth to note that the type high circular dichroism semiconductor laser work not only in the visible wavelength. By integrating the spiral nanowire cavity with the high quality gain materials such as GaAs or InP, the small footprint high CD light sources could be also realize in infrared (IR) wavelength region, which is more suitable in biological applications.

## Summary

In conclusion, we fabricated Al-GaN NWR lasers which were folded into the L- and R-hand chiral structures for high-CD laser outputs at UV wavelengths. The degrees of circular polarizations of these chiral lasers were characterized with a circular-polarization analyzer. The dissymmetry factors of the L- and R-chiral lasers were approximately 1.4 and −1.6, respectively, and the devices have a footprint of only a few micrometers. The results showed that Al-GaN chiral NWR cavities with only 5 periods were sufficient for lasing and high dissymmetry factors. These compact lasers with high dissymmetry factors are ideal for future applications related to circularly polarized photons.

## Method

### Modeling

The finite-element method (FEM) was applied to design the metal-coated GaN chiral structures, setting the high optical confinement around the gain spectrum of the GaN epitaxial layer in UV wavelength region. The simulation model of the metal-coated chiral structures consisted of aluminum, Si_3_N_4_, and undoped GaN. The refractive indices of the materials of the cavity structure have been discussed and included in the supplementary document. We adhered to the above structure design to construct the left and right GaN spiral nanowires. We then set the 30-nm dielectric layer and the 50-nm metal layer and established a perfectly matched layer around the GaN spiral nanowires to absorb redundant signals, which might reflect back to the metal-coated chiral structures, simulating their electric-field mode profile. Finally, we determined a circular oscillation mode in the designed structures.

The modal fields in Fig. 5(b,c) and corresponding joint quality factors *Q*_joint_ are calculated based on a frequency-domain computational scheme aimed at active photonioc devices^44^,^45^. The scheme incorporates the effect of sources and gain. The core of this method is a generalized eigenvalue (GE) problem originating from Maxwell’s equation: 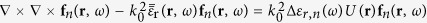, where **f**_*n*_(**r**, *ω*) is the field profile of mode *n* at a given frequency *ω*; *k*_0_ is the propagation constant in vacuum; 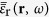 is the permittivity tensor corresponding to the resonance structure; *U*(**r**) is an indicator function which is unity in the active region but vanishes elsewhere; and Δ*ε*_r,*n*_(*ω*) is the eigenvalue representing the permittivity variation required for self-oscillation (lasing) of the mode. The resonance frequency *ω*_*n*_ of mode *n* is the frequency which minimizes the modulus 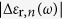, namely, 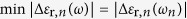. After *ω*_*n*_ is obtained, one can estimate the quality factor as 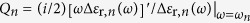, where the prime (′) means the derivative with respect to *ω*.

In the presence of bending, we utilize FEM to implement the GE problem in the cylindrical coordinate with the ansatz 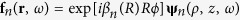, where *R* is the local curvature radius; *β*_*n*_(*R*) is a real number describing the local propagation along the azimuthal direction; and **ψ**_*n*_(*ρ*, *z*, *ω*) is the modal field excluding the azimuthal dependence. The active region is set to the GaN waveguide core. To fix *β*_*n*_(*R*) at a given radius *R*, we first set *ω* to the experimental lasing frequency (equivalent to 364 nm) and pick up *β*_*n*_(*R*) that minimizes the modulus |Δ*ε*_r,*n*_(*ω*)| of the target mode. After *β*_*n*_(*R*) is determined, we then vary *ω* to minimize |Δ*ε*_r,*n*_(*ω*)| and obtain *ω*_*n*_, which is typically very close to the experimental lasing frequency. The joint quality factor *Q*_joint_ = *Q*_*n*_ is then estimated with the aforementioned procedure. This quality factor carries the information on the bending and leakage losses corresponding to the local radius *R*. The magnitudes of radial components 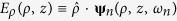 at different radii 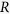 are then shown as the modal fields in Fig. 5(b,c).

### Characterization

The metal-coated GaN spiral nanowire cavities were characterized by a micro-photoluminescence (μ-PL) system. The devices were optically pumped using a frequency-tripled Nd:YVO_4_ 355-nm pulsed laser at room temperature. The details of the characterization are described in the supplementary information.

## Additional Information

**How to cite this article**: Liao, W.-C. *et al.* Optimized Spiral Metal-Gallium-Nitride Nanowire Cavity for Ultra-High Circular Dichroism Ultraviolet Lasing at Room Temperature. *Sci. Rep.* **6**, 26578; doi: 10.1038/srep26578 (2016).

## Supplementary Material

Supplementary Information

## References

[b1] Valev V. K., Baumberg J., Sibilia C. & Verbiest T. Chirality and Chiroptical Effects in Plasmonic Nanostructures: Fundamentals, Recent Progress, and Outlook. Advanced Materials. 25, 2517–2534, doi: 10.1002/adma.201205178 (2013). 23553650

[b2] Farshchi R., Ramsteiner M., Herfort J., Tahraoui A. & Grahn H. Optical Communication of Spin Information between Light Emitting Diodes. Applied Physics Letters. 98, 162508, doi: 10.1063/1.3582917 (2006).

[b3] Sherson J. F. *et al.* Quantum Teleportation between Light and Matter. Nature 443, 557–560, doi: 10.1038/nature05136 (2006). 17024089

[b4] Wagenknecht C. *et al.* Experimental Demonstration of a Heralded Entanglement Source. Nature Photonics 4, 549–552 , doi: 10.1038/nphoton.2010.123 (2010).

[b5] Schadt M. Liquid Crystal Materials and Liquid Crystal Displays. Annual Review of Materials Science 27, 305–379, doi: 10.1146/annurev.matsci.27.1.305 (1997).

[b6] Atkins P. W. & DePaula J. The Elements of Physical Chemistry. (Oxford University Press, 2005).

[b7] Solomon E. I., Lever A., Czernuszewicz R. & Spiro T. Inorganic Electronic Structure and Spectroscopy. Vol 1: Methodology (John Wiley and Sons, 1999).

[b8] Rau H. Asymmetric Photochemistry in Solution. Chemical Reviews 83, 535–547, doi: 10.1021/cr00057a003 (1983).

[b9] Inoue Y. Asymmetric Photochemical Reactions in Solution. Chemical Reviews 92, 741–770, doi: 10.1021/cr00013a001 (1992).

[b10] Krenn H., Zawadzki W. & Bauer G. Optically Induced Magnetization in a Dilute Magnetic Semiconductor: Hg1-xMnxTe. Physical Review Letters 55, 1510–1513, doi: 10.1103/PhysRevLett.55.1510 (1985). 10031842

[b11] Awschalom D., Warnock J. & Von Molnar S. Low-Temperature Magnetic Spectroscopy of a Dilute Magnetic Semiconductor. Physical Review Letters 58, 812, doi: 10.1103/PhysRevLett.58.812 (1987). 10035043

[b12] Maier S. A. Plasmonic Fundamentals and Applications. (Springer, 2007).

[b13] Smith D. R., Pendry J. B. & Wiltshire M. C. K. Metamaterials and Negative Refractive Index. Science 305, 788–792, doi: 10.1126/science.1096796 (2004). 15297655

[b14] Barnes W. L., Dereux A. & Ebbesen T. W. Surface Plasmon Subwavelength Optics. Nature 424, 824–830, doi: 10.1038/nature01937 (2003). 12917696

[b15] Schurig D. D. R. *et al.* Metamaterial Electromagnetic Cloak at Microwave Frequencies. Science 10, 977–980, doi: 10.1126/science.1133628 (2006). 17053110

[b16] Yu N. *et al.* A Broadband, Background-free Quarter-wave Plate Based on Plasmonic Metasurfaces. Nano Letters 12, 6328–6333, doi: 10.1021/nl303445u (2012). 23130979

[b17] Tang Y., Sun L. & Cohen A. E. Chiroptical Hot Spots in Twisted Nanowire Plasmonic Oscillators. Applied Physics Letters 102, 043103, doi: 10.1063/1.4789529 (2013).

[b18] Yeom B. *et al.* Chiral Plasmonic Nanostructures on Achiral Nanopillars. Nano Letters 13, 5277–5283, doi: 10.1021/nl402782d (2013). 24111695

[b19] Maksimov A. *et al.* Circularly Polarized Light Emission from Chiral Spatially-structured Planar Semiconductor Microcavities. Physical Review B 89, 045316, doi: 10.1103/PhysRevB.89.045316 (2014).

[b20] Chen J. Y., Wong T. M., Chang C. W., Dong C. Y. & Chen Y. F. Self-polarized Spin-Nanolasers. Nature Nanotechnology 9, 845–850, doi: 10.1038/nnano.2014.195 (2014). 25240673

[b21] Konishi K. *et al.* Circularly Polarized Light Emission from Semiconductor Planar Chiral Nanostructures. Physical Review Letters 106, 057402, doi: 10.1103/PhysRevLett.106.057402 (2011). 21405435

[b22] Cui Y., Kang L., Lan S., Rodrigues S. & Cai W. Giant Chiral Optical Response from a Twisted-arc Metamaterial. Nano Letters 14, 1021–1025, doi: 10.1021/nl404572u (2014). 24422639

[b23] Drezet A., Genet C., Laluet J. Y. & Ebbesen T. W. Optical Chirality without Optical Activity: How Surface Plasmons Give a Twist to Light. Optics Express 16, 12559–12570, doi: 10.1364/OE.16.012559 (2008). 18711492

[b24] Bachman K. *et al.* Spiral Plasmonic Nanoantennas as Circular Polarization Transmission Filters. Optics Express. 20, 1308–1319, doi: 10.1364/OE.20.001308 (2012). 22274476

[b25] Lin J. *et al.* Polarization-Controlled Tunable Directional Coupling of Surface Plasmon Polaritons. Science 340, 331–334, doi: 10.1126/science.1233746 (2013). 23599488

[b26] Chen C. F. *et al.* Creating Optical Near-Field Orbital Angular Momentum in a Gold Metasurface, Nano Letters. 15, 2746–2750, doi: 10.1021/acs.nanolett.5b00601 (2015). 25798810

[b27] Tsai W. Y., Huang J. S. & Huang C. B. Selective trapping or rotation of isotropic dielectric micro-particles by optical near field in a plasmonic Archimedes spiral. Nano Letters 14, 547–552, doi: 10.1021/nl403608a (2014). 24392638

[b28] Hill M. T. *et al.* Lasing in Metallic-coated Nanocavities. Nature Photonics 1, 589–594, doi: 10.1038/nphoton.2007.171 (2007).

[b29] Noginov M. A. *et al.* Demonstration of a Spaser-based Nanolaser. Nature 460, 1110–1112, doi: 10.1038/nature08318 (2009). 19684572

[b30] Yu K., Lakhani A. & Wu M. C. Subwavelength Metal-optic Semiconductor Nanopatch Lasers. Optics Express 18, 8790–8799, doi: 10.1364/OE.18.008790 (2010). 20588723

[b31] Nezhad M. P. *et al.* Room-temperature Subwavelength Metallo-dielectric Lasers. Nature Photonics 4, 395–399, doi: 10.1038/nphoton.2010.88 (2010).

[b32] Lu Y. J. *et al.* Plasmonic Nanolaser Using Epitaxially Grown Silver Film. Science. 337, 450–453, doi: 10.1126/science.1223504 (2012). 22837524

[b33] Ding K. & Ning C. Z. Metallic Subwavelength-cavity Semiconductor Nanolaser. Light: Science and Applications 1, e20, doi: 10.1038/lsa.2012.20 (2012).

[b34] Oulton R. F. *et al.* Plasmon Lasers at Deep Subwavelength Scale. Nature 461, 629–632, doi: 10.1038/nature08364 (2009). 19718019

[b35] Yang A. *et al.* Real-time Tunable Lasing from Plasmonic Nanocavity Arrays. Nature Communications 6, 6939, doi: 10.1038/ncomms7939 (2015). PMC4411284 25891212

[b36] Wang Y. G. *et al.* Lasing in Metal-coated GaN Nanostripe at Room Temperature. Applied Physics Letters 98, 131110, doi: 10.1063/1.3572023 (2011).

[b37] Chen K. J., Hsu W. H., Liao W. C., Shih M. H. & Kuo H. C. Lasing Characteristics of Metal-coated GaN with Grating Structure at Room Temperature. IEEE Journal of Selected Topics in Quantum Electronics 21, 4600105, doi: 10.1109/JSTQE.2014.2336536 (2015).

[b38] Bozhevolnyi S. I., Volkov V. S., Devaux E., Laluet J. Y. & Ebbesen T. W. Channel Plasmon Subwavelength Waveguide Components Including Interferometers and Ring Resonators. Nature 440, 508–511, doi: 10.1038/nature04594 (2006). 16554814

[b39] Wang W., Yang Q., Fan F., Xu H. & Wang Z. L. Light Propagation in Curved Silver Nanowire Plasmonic Waveguides. Nano Letters 11, 1603–1608, doi: 10.1021/nl104514m (2011). 21410216

[b40] Holmgaard K. T. *et al.* Bend-and Splitting Loss of Dielectric-loaded Surface Plasmon-polariton Waveguides. Optics Express 16, 13585–13592, doi: 10.1364/OE.16.013585 (2008). 18772968

[b41] Dikken D. J., Spasenović M., Verhagen E. & Oosten D. Kuipers, L. Characterization of Bending Losses for Curved Plasmonic Nanowire Waveguides. Optics Express 18, 16112–16119, doi: 10.1364/OE.18.016112 (2010). 20720996

[b42] Kim M. W. & Ku P. C. Lasing in a Metal-clad Microring Resonator. Applied Physics Letters 98, 131107, doi: 10.1063/1.3573818 (2011).

[b43] Wang Y. G. *et al.* Room Temperature Lasing with High Group Index in Metal-coated GaN Nanoring. Applied Physics Letters 99, 251111, doi: 10.1063/1.3671648 (2011).

[b44] Chang S. W. Full frequency-domain approach to reciprocal microlasers and nanolasers-perspective from Lorentz reciprocity. Optics Express 19, 21116–21134, doi: 10.1364/OE.19.021116 (2011). 22108963

[b45] Chiang P. J. & Chang S. W. Frequency-domain formulation of photonic crystals using sources and gain. Optics Express 21, 1972–1985, doi: 10.1364/OE.21.001972 (2013). 23389178

[b46] Moirangthem R. S. *et al.* Optical cavity modes of a single crystalline zinc oxide microsphere. Optics Express 21, 3010–3020, doi: 10.1364/OE.21.003010 (2013). 23481759

