# Supplementary material for: Optimized Spiral Metal-Gallium-Nitride Nanowire Cavity for Ultra-High Circular Dichroism Ultraviolet Lasing at Room Temperature
Source: Sci Rep. 2016 May 25;6:26578. doi: 10.1038/srep26578 (PMC4879524; doi:10.1038/srep26578)
Supplement: Supplementary Information [file srep26578-s1.pdf]

Supplementary Information

Optimized Spiral Metal-Gallium-Nitride Nanowire Cavity for Ultra-High Circular Dichroism Ultraviolet Lasing at Room Temperature

Wei-Chun Liao[1], Shu-Wei Liao[1], Kuo-Ju Chen[1], Yu-Hao Hsiao[1], Shu-Wei Chang[1,2], Hao-Chung Kuo[1] , and Min-Hsiung Shih[1,2,3*]

[1]*Department of Photonics and Institute of Electro-Optical Engineering, National Chiao Tung University (NCTU), Hsinchu 30010, Taiwan*

[2]*Research Center for Applied Sciences (RCAS), Academia Sinica, Taipei 11529, Taiwan*

[3]*Department of Photonics, National Sun Yat-sen University (NSYSU), Kaohsiung 80424, Taiwan*

**E-mail address: mhshih@gate.sinica.edu.tw*

1) The material indices used in the simulation

The hybrid surface plasmon waves around the interfaces of *GaN* nanowire and the aluminum (*Al*) layer play an important role to achieve a high-Q and low loss cavity. This leads to the room temperature lasing action within such small footprint. To understand the optical modes in the nanocavity, we performed the simulation with the finite-element method (FEM), and calculated the optical mode profiles in the UV lasing wavelength.

The two spectra in Figure S1 are the real part (*n*) and image part (*k*) of refractive index of aluminum (*Al*) metal layer used in the simulation. Figure S2 show refractive index of silicon nitride (*SiNx*) layer. We only consider the real part of SiNx refractive index since the absorption of *SiNx* is small enough to ignore within the lasing wavelength region. Figure S3 shows the real part (*n*) and image part (*k*) of refractive index of gallium nitride (*GaN*) layer. We obtained the refractive indexes of undoped GaN and the aluminum layer established by Rakic et al. [R1] and Peng and Piprek [R2].

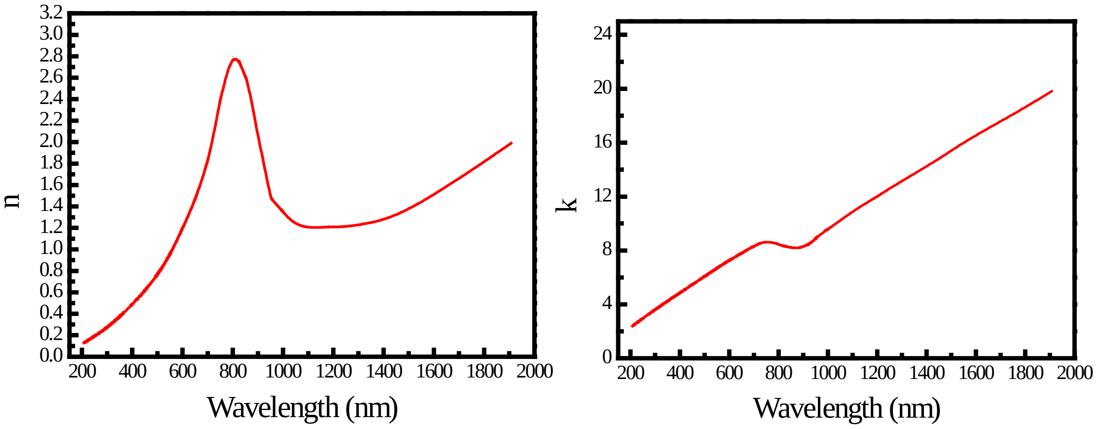

n k

Figure S1. The real part and image part of refractive index of aluminum (Al) metal layer used in the simulation.

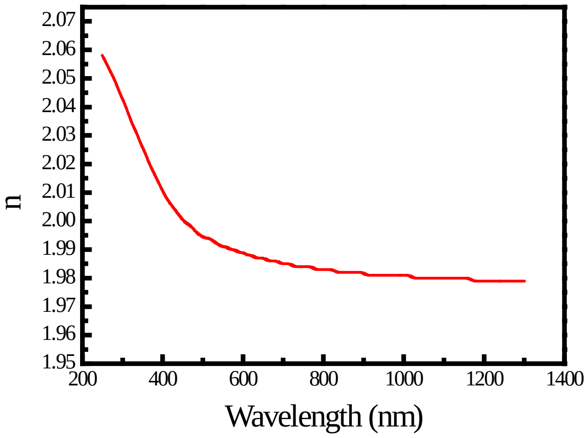

Figure S3. The refractive index of the silicon nitride (SiNx) layer used in the simulation.

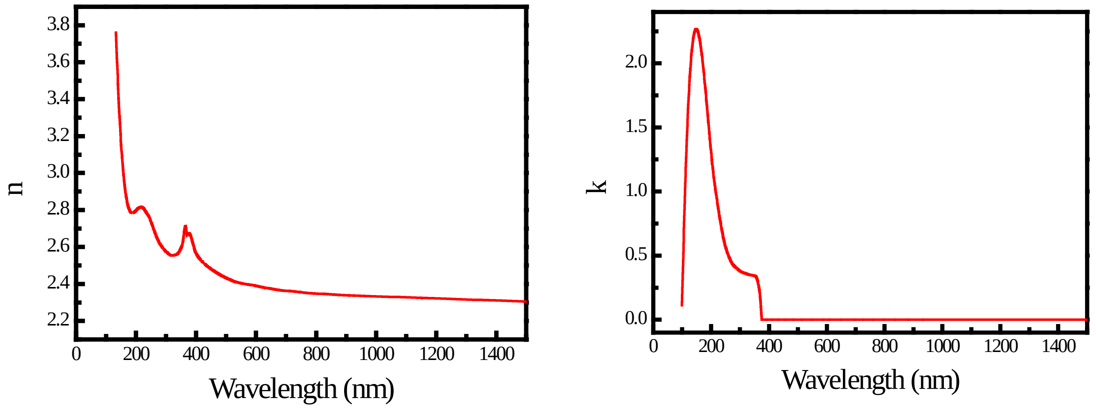

n k

Figure S3. The real part and image part of refractive index of the undoped gallium nitride (GaN) material in the spiral nanowire cavity simulation.

References:

[R1] A. D. Rakić, A. B. Djurišic, J. M. Elazar, and M. L. Majewski, *Appl. Opt.* **37**, 5271-5283 (1998).

[R2] T. Peng and J. Piprek, *Electron. Lett.* **32**, 2285 (1996).
